# Titanium dioxide nanoparticles stimulate sea urchin immune cell phagocytic activity involving TLR/p38 MAPK-mediated signalling pathway

**DOI:** 10.1038/srep14492

**Published:** 2015-09-28

**Authors:** Annalisa Pinsino, Roberta Russo, Rosa Bonaventura, Andrea Brunelli, Antonio Marcomini, Valeria Matranga

**Affiliations:** 1 Consiglio Nazionale delle Ricerche, Istituto di Biomedicina e Immunologia Molecolare “A. Monroy”, Via Ugo La Malfa 153, 90146 Palermo, Italy; 2 Dipartimento di Scienze Ambientali, Informatica e Statistica, Università Ca’ Foscari Venezia, Calle Larga S. Marta 2137, 30123 Venezia, Italy

## Abstract

Titanium dioxide nanoparticles (TiO_2_NPs) are one of the most widespread-engineered particles in use for drug delivery, cosmetics, and electronics. However, TiO_2_NP safety is still an open issue, even for ethical reasons. In this work, we investigated the sea urchin *Paracentrotus lividus* immune cell model as a *proxy to humans*, to elucidate a potential pathway that can be involved in the persistent TiO_2_NP-immune cell interaction *in vivo*. Morphology, phagocytic ability, changes in activation/inactivation of a few mitogen-activated protein kinases (p38 MAPK, ERK), variations of other key proteins triggering immune response (Toll-like receptor 4-like, Heat shock protein 70, Interleukin-6) and modifications in the expression of related immune response genes were investigated. Our findings indicate that TiO_2_NPs influence the signal transduction downstream targets of p38 MAPK without eliciting an inflammatory response or other harmful effects on biological functions. We strongly recommend sea urchin immune cells as a new powerful model for nano-safety/nano-toxicity investigations without the ethical normative issue.

Nanotechnology is one of the most active research areas in our contemporary society. The presence of engineered nano-objects and their aggregates and agglomerates (NOAA) in many consumer products has attracted a growing scientific concern on their possible effects on the environment and human health^1^,^2^. Due to their low dissolution rate, high surface area, anti-corrosive and photo-catalytic properties, titanium dioxide nanoparticles (TiO_2_NPs) are one of the most widespread particles for drug delivery, antibacterial materials, cosmetics, sunscreens, electronics^3^. These same features may present unique bioactive properties promoting or preventing human health; in fact, the growing number of published studies confirm the high level of interest concerning the TiO_2_NP safety^4^. However, a comprehensive understanding of the TiO_2_NP safety has not been achieved yet, even due to ethical reasons. In recent years, the European Partnership for Alternative Approaches to Animal Testing (EPAA) is committed to pooling knowledge and resources to accelerate the development, validation and acceptance of alternative approaches to promote the replacement, reduction and refinement (3Rs) of animal use in regulatory testing. In this context, the sea urchin provides an attractive and alternative *proxy to human* non-mammalian model for exploring the safety of TiO_2_NPs as it shows: i) no ethical normative problems, in full respect of the 3Rs criteria of the EPAA; ii) ease and responsiveness to experimental manipulation; iii) evident homology with human genes^5^. The sea urchin is a marine invertebrate in the lineage leading to the vertebrates and humans^6^. It possesses an extraordinary and singular adaptive capacity to environmental changes, due to its responsive immune system, which provides protection, robustness, and molecular plasticity, both in the adult and in the embryonic lives^5^,^7^,^8^. Analyses of the sea urchin genome revealed an exceptional expansion and diversification of several classes of host sensors genes, collectively named *pattern recognition molecules*, part of which are closely related to human homologs^7^,^9^,^10^. Sea urchin specialized immune cells protect the adult organism as a heterogeneous population of freely moving cells localized to distinct compartments in the body, including the perivisceral coelomic cavities and the water-vascular system. At least three morphologically and likely functionally distinct circulating immune cell types are found in the coelomic fluid (a fluid with functions similar to the blood of higher animals) of the sea urchin *Paracentrotus lividus*, which include phagocytes, amoebocytes and vibratile cells^5^,^11^.

Mitogen-activated protein kinases (MAPKs) are a group of serine/threonine protein kinases mediating a wide range of cellular processes, including cell proliferation, cell differentiation, apoptosis and immune response^12^. The members of the major MAPK subfamilies, namely the p38 mitogen-activated protein kinase (p38 MAPK), the Jun N-terminal kinase (JNK) and the extracellular signal-regulated kinase (ERK), establish interconnected signal transduction cascades activated by external stimuli, such as stress, growth factors, cytokines^13^. In mammalian species, MAPKs are involved in all aspects of immune responses, from the beginning phase of innate immunity, to activation of adaptive immunity, and to cell death when immune response is concluded^13^. The signals that lead to MAPK activation are usually elicited at the cell surface mainly by a broad spectrum of membrane-bound receptors known as pattern recognition receptors (PRRs), which are involved in the initiation, promotion and execution of immune responses^14^. Among the PRRs, the family of Toll-like receptors (TLRs) have been studied most extensively^15^. Recently, several studies indicated that TLRs are involved in cellular uptake and immune response to TiO_2_NP exposure in human cell lines and mouse embryonic cell lines, although relatively little is known about such interactions^16^,^17^,^18^,^19^,^20^. For example, authors support the evidence that among these receptors, in human hepatocellular carcinoma cells exposed to TiO_2_NPs, the TLR3 attenuates protein denaturation and heat shock response (HSP70) and the TLR4 mediates uptake and inflammation^17^. After TLR activation, the intracellular signal transduction involving MAPKs can be propagated through a phosphorylation cascade that eventually leads to changes in gene expression and/or protein activity^14^. However, the current knowledge on the signalling that mediates the interaction between TiO_2_NPs and immune cells remain scant, especially in marine animal models.

In this study, we focused on the molecular basis of the effects of TiO_2_NPs on sea urchin immune cell behaviour. Since phagocytosis is an early line of defence against host invasion, and phagocytes are the most abundant type of immune cells present in the body fluid of the sea urchin, we postulated that TiO_2_NPs are able to elicit a receptor-mediated phagocytic mechanism involving one or more MAPKs. To test this hypothesis we analysed immune cell morphology, phagocytic ability, changes in activation/inactivation of a few MAPKs (p38 MAPK, ERK), variations in the levels of other key proteins triggering immune response (TLR4-like, HSP70, Interleukin-6) and the expression of related immune response genes.

Our findings indicate that TiO_2_NPs influence the signal transduction downstream targets of p38 MAPK without eliciting an inflammatory response or other harmful effects on biological functions. These results provide new insights into the molecular mechanisms and the factors involved in the progression of Echinoderm immune response after TiO_2_NP *in vivo* exposure, and provide intriguing suggestion concerning the use of the sea urchin immune cells as a new powerful tool for nano-safety/nano-toxicity investigations.

## Results

### Animal behaviour, cell viability and density

**Figure 1:**
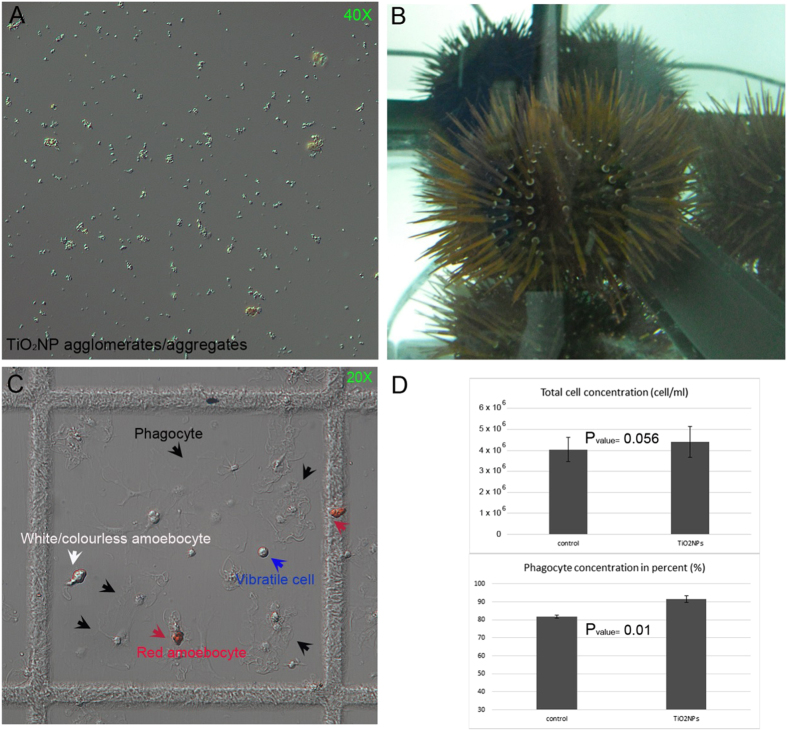
TiO_2_ nanoparticles, adult sea urchins and immune cells under the microscope. (**A**) TiO_2_NP agglomerates/aggregates of different sizes observed 60 minutes after suspension in ASW. (**B**) *Paracentrotus lividus* sea urchins after 24-hour exposure to TiO_2_NP suspension (1 μg/ml concentration). (**C**) Living immune cells in a Fast-Read chamber harvested as a total cell population by bleeding sea urchins exposed for 24 hour through a cut in the peristomal membrane. (**D**) Graphic representation of the total cell concentration (cell/ml, upper graph) and the number of phagocytes (in percent, lower graph). Cell types are indicated by captions of different colors and corresponding pointing arrows: red amoebocyte (red arrows), white/colourless amoebocyte (white arrow), phagocyte (black arrows), and vibratile cell (blue arrow). Images were captured by Zeiss Axioskop 2 Plus microscope (Zeiss, Arese, Italy).

TiO_2_NPs suspended in several salt water media at different concentrations were previously characterized by a combination of analytical techniques, including transmission electron microscopy (TEM) and dynamic light scattering (DLS)^21^. Pristine TiO_2_NP powder exhibited a size distribution ranging from 10 to 65 nm, and a moderately irregular and semi-spherical shape, classified as mesoporous NPs^21^ (Table 1). TiO_2_NPs appeared as compact, large agglomerates/aggregates of 350 ± 41 nm and 466 ± 9 nm, at 0.2 and 25 hours after suspension in the salt water media (1 μg/ml nominal concentration)^21^. Regardless of the initial concentrations used, particles morphology and size of TiO_2_NPs dispersed in artificial seawater (ASW) were inspected by optical microscopy after 60 minutes. A representative image of big agglomerates/aggregates of different dimensions (μm) suspended in ASW (1 μg/ml) is shown in Fig. 1A.

Exposure conditions (1–5 μg/ml, 24 hours, 30-gauge syringe needle) were chosen taking into consideration: i) the behaviour of TiO_2_NPs in salt water media^21^; ii) the results obtained from studies performed in others marine organisms^22^,^23^ and iii) the known immune-activation of the Echinoderm immune cells upon injection^24^,^25^.

The TiO_2_NP suspensions were injected into the sea urchin body cavity of groups of 3 sea urchins in order to expose immune cells *in vivo*. Sea urchin physiological conditions were monitored in terms of: general animal motility, active ejection or passive loss of spines, tube feet clamping force, induction of gamete spawning and expulsion of fecal matters. No noticeable pathological state was observed at both concentrations used (1–5 μg/ml) over 24 hours exposure. Animals displayed a typical locomotion capacity, regular movement of the spines and tube feet, characteristic adhesion ability. In addition, they did not start to drop or loose spines, as well as to excrete in excess. Since observations on animal behavior did not show any adverse effect at the tested concentrations, only the lowest concentration (i.e. 1 μg/ml), the most used in marine models, was used for the following morphological and molecular analyses performed 1-day after exposure (Fig. 1B). As three main different cell types of freely circulating immune cells are present in *Paracentrotus lividus* (phagocytes, amoebocytes and vibratile cells)^5^, a morphological analysis of viable cells on microscopical slides was performed, as shown in Fig. 1C, and determined their relative quantities in triplicate samples. TiO_2_NPs did not significantly influence the number of circulating cells as total cell population in exposed animals, but produced a slight increase (10%) in the relative number of phagocytes (P_value_ = 0.01) (Fig. 1D).

### TiO_2_ nanoparticles stimulate phagocytic activity of sea urchin immune cells and increase a Toll-like receptor (*Pl-TLR*) mRNA and protein levels

**Figure 2:**
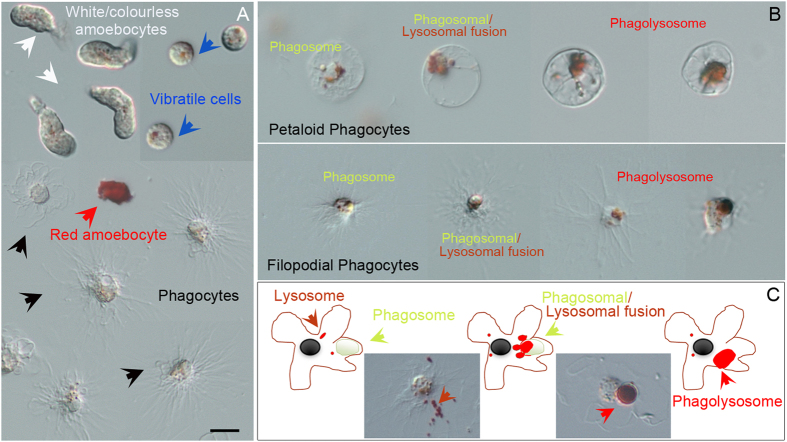
Neutral Red for live cell imaging of the phagocytic activity of exposed immune cells. (**A**) The pH-indicator dye NR is concentrated in lysosomes forming typical small red vesicles within all three different cell types of freely circulating immune cells present in *Paracentrotus lividus* (phagocytes, amoebocytes and vibratile cells), most evident in the vibratile cells. Cell types are indicated by captions of different colors and corresponding pointing arrows as described in Fig. 1. (**B**) Petaloid and filopodial phagocytes show areas of high lysosomal and phagocytic activity in which NR became concentrated. (**C**) Schematic model and demonstrative pictures representing an immune cell undergoing phagosomal maturation. Arrows indicate lysosomes (orange arrows), phagosomal/lysosomal fusion (yellow arrows), phagolysosome (red arrows). Bar 5 μm.

In order to investigate the sea urchin immune cell machinery implicated in TiO_2_NP recognition and interaction, live cell imaging with specific dyes was used, such as Neutral Red (NR) and 3, 3′-dihexyloxacarbocyanine iodide (DiOC_6_), to detect the involvement of subcellular organelles. Specifically, the pH-indicator dye NR to detect lysosomal pH dynamics (intracellular acidification) and the membrane-potential dye DiOC_6_ to monitor lysosomal internal membrane stability. After a 15 min *ex vivo* exposure, NR became concentrated in lysosomes, forming typical small red vesicles within all cell types, more evident in the vibratile cells (Fig. 2A). The progression of the *bona fide* phagosomal maturation accompanied by luminal acidification was detected in a number of phagocytic cells (25% to 30% of phagocytes), capable of phagocytizing in both their petaloid and filopodial shapes (Fig. 2B). The NR immune-localization revealed that both the two types of phagocytic cells presented different degree of vesicle maturation, such as: i) early phagosomes (4.1%, white vesicles); ii) phagosome which are fusing with lysosomes (19.3%); iii) phagolysosome (3.7%).

Only a few phagocytes showed the NR leaking (damaged lysosomes are unable to retain the dye) that reflected the efflux of lysosomal contents into the cytosol following lysosomal membrane disaggregation (not shown), which demonstrate that TiO_2_NPs did not affect lysosomial function of cells.

**Figure 3:**
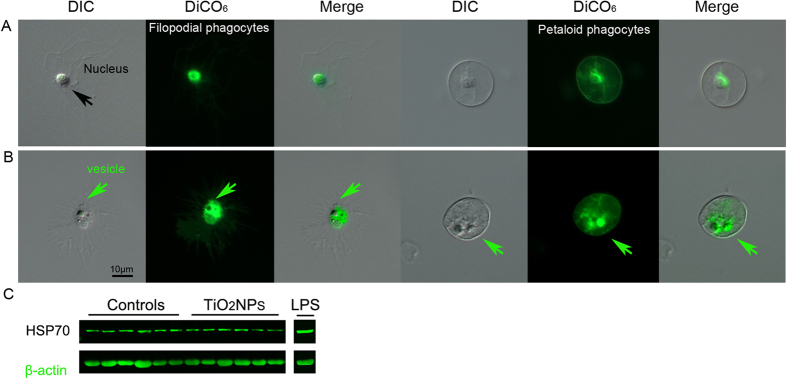
Dihexyloxacarbocyanine iodide to monitor lysosomal internal membrane stability of TiO_2_ exposed immune cells. (**A**) The green fluorescent membrane dye DiOC_6_ is detected within the cytoplasm, as a dense signal around the nuclei of both the non-activate filopodial and petaloid phagocytes. (**B**) A few cells showed a growing network of vesicles (green arrows), validating a phagocytic activity in progress. (**C**) Representative image of the HSP70 levels of 6 control specimens, 6 specimens exposed to TiO_2_NP and 1 to LPS, evaluated by immunoblotting. Contrary to LPS exposure, TiO_2_NP exposure do not stimulate the activation of the HSP70-dependent stress response in the sea urchin immune cells.

*Ex vivo* DiOC_6_ labelling, showed a discrete fluorescence within the cytoplasm, a dense signal around the nuclei of all immune cells morphotypes, suggesting that TiO_2_NPs did not affect internal membrane polarization of the trans-Golgi/endoplasmic reticulum (ER) compartments, as well as other vesicle membranes. Interestingly, several phagocytes showed a growing network of vesicles, confirming a phagocytic activity in progress (Fig. 3). Our results highlighted a general healthy state of immune cells, as TiO_2_NP exposure was not perceived as a stress, established by the fact that it did not stimulate the activation of the HSP70-dependent stress response (Fig. 3C).

**Figure 4:**
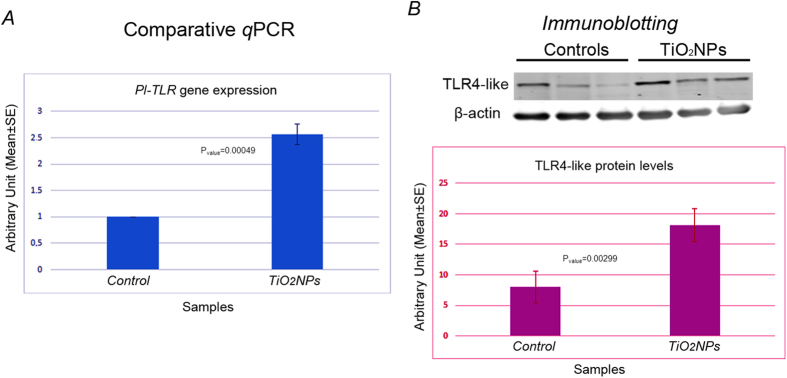
TiO_2_ nanoparticles stimulate a member of the Toll-like receptor family. (**A**) Levels of expression of *Pl*-*TLR* gene analysed by comparative *q*PCR with total RNA isolated from control (injected only with ASW) and exposed immune cells. Levels are expressed in arbitrary units as fold increase compared to controls assumed as 1, using the endogenous gene *Pl-Z12-1* for normalization. Each bar represents the mean of three independent experiments ±SE. (**B**) Representative images of the TLR4-like protein levels of 3 control specimens and 3 specimens exposed to TiO_2_NPs evaluated by immunoblotting. Histograms represent the means of the three independent experiments (18 specimens obtained from 3 independent experiments) ±SE after normalization with actin levels.

The expression levels of a member of the Tlr family were measured by real-time *q*PCR and the TLR4-like protein levels were evaluated by immunoblotting upon the *in vivo* exposure of sea urchins to 1 μg/ml TiO_2_NPs. After a 1-day exposure, immune cells showed levels of expression of the *Pl-TLR* gene 2.6-fold higher than measured in control cells (collected from sea urchin receiving only an ASW injection) (P_value_ = 0.00049) (Fig. 4A). A similar increase in the levels of the TLR4-like protein (P_value_ = 0.00299) was found (Fig. 4B). Analogous results were obtained in cells exposed to 5 μg/ml nominal concentration (not shown). Immunoblotting was performed by using an anti-TLR4 antibody that labelled a band of about 70 kDa.

### TiO_2_ nanoparticles affect p38 MAPK- but not ERK- mediated signalling pathway

**Figure 5:**
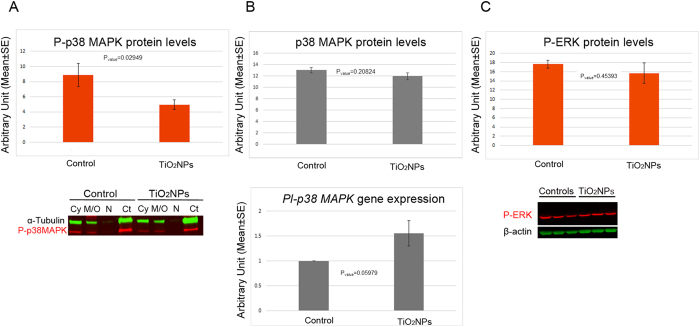
TiO_2_ nanoparticles affect phosphorylation levels of the p38 MAPK. (**A**) Immunoblotting analysis with anti-P-p38 MAPK on total cell lysates (upper panel) and subcellular fractions (lower panel) of sea urchin immune cells exposed to TiO_2_NPs, shows a significant reduction (50%) in the in the levels of the phosphorylated form of p38 MAPK. (**B**) Immunoblotting analysis with anti-p38 MAPK on total cell lysates of sea urchin immune cells (upper panel) and comparative *q*PCR analysis of *Pl-p38 MAPK* gene expression (lower panel). (**C**) Immunoblotting analysis with anti-P-ERK (upper panel) and representative image of 3 control specimens, and 3 specimens exposed to TiO_2_NPs (lower panel). Histograms represent the means of the three independent experiments ±SE after normalization with tubulin or actin levels for the proteins and *Pl-Z12-1* gene for the gene expression. Cy: cytosol; M/O: membrane/organelles; N: nuclei; Ct: cytoskeleton.

MAPKs are key components of the signalling regulating the innate immune response triggered by the TLRs^14^. Since TLR4 engagement is activating both ERK and p38 MAPK downstream signalling pathways, ultimately resulting in the generation of a pro-inflammatory response, the activation/inactivation was investigated to clarify whether a potential involvement in a hypothetical immune response stimulated by TiO_2_NPs may have occurred. The activation of both ERK and p38 MAPK was analysed by immunoblotting performed on the total cell lysates (Fig. 5A). Control immune cells (collected from sea urchin receiving only ASW injection) showed considerable constitutive levels of phospho-p38 MAPK (P-p38 MAPK). On the contrary, TiO_2_NP-exposed cells displayed a significant reduction (50%) in the P-p38 MAPK levels (P_value_ = 0.02949) (Fig. 5A upper panel). Subcellular fractionation into cytosol (Cy), membranes/organelles (M/O), nuclei (N), and cytoskeleton (Ct) of the sea urchin immune cells confirmed results obtained using total cell lysates. P-p38 MAPK was found localized in all cellular compartments of both control and exposed cells, with a major localization in the Ct of the controls (Fig. 5A lower panel). On the contrary, no significant differences in the levels of the non-phosphorylated (non-activated) p38 MAPK form (P_value_ = 0.20824) (Fig. 5B upper panel) as well as the *Pl*-*p38 MAPK* mRNA (P_value_ = 0.05979) (Fig. 5B lower panel) were observed in controls and exposed cells. In addition, TiO_2_NP exposure was not effective in inhibiting or enhancing phosphorylation levels of ERK (Fig. 5C). MAPK activation/inactivation was also investigated in cells exposed to 5 μg/ml nominal concentration and results were similar to those obtained with the lower concentration (not shown).

### TiO_2_ nanoparticles affect signal transduction downstream to p38 MAPK

To focus on the role of p38 MAPK, as signal transduction mediator of the immune response through the activation of specific transcription factors and/or other kinases, the expression of the nuclear factor kappa B (NF-*k*B) and the Jun transcription factor (Jun), which are known as two of the most sensitive transcription factors associated with inflammation^26^,^27^ were examined.

**Figure 6:**
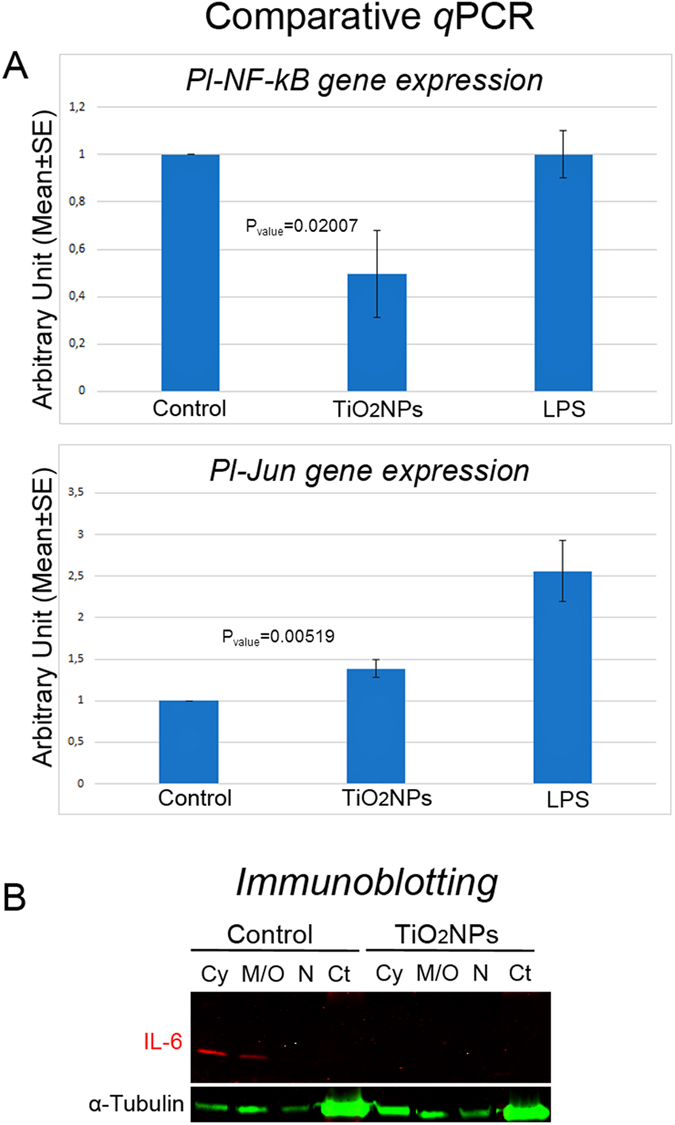
Signal transduction downstream to p38 MAPK in response to TiO_2_ nanoparticles. (**A**) Comparative qPCR analysis show levels of expression of *Pl*-*NF-kB* gene 2-fold lower than those measured in controls (injected only with ASW) (upper panel), while the transcript of *Pl*-*Jun* showed a weak increase in its levels (lower panel). Levels are expressed in arbitrary units as fold increase compared to controls assumed as 1, using the endogenous gene *Pl-Z12-1* for normalization. Results were compared to those obtained in response to LPS (2 μg/ml in ASW, 24 h): in this case, the levels of expression of *Pl-NF-kB* gene were found comparable to controls while the transcript of *Pl-Jun* was found strongly over-expressed (2.56 ± 0.71-fold). Each bar represents the mean of three independent experiments ±SE. (**B**) Immunoblotting analysis with anti-IL6 subcellular fractions of sea urchin immune cells. Cy: cytosol; M/O: membrane/organelles; N: nuclei; Ct: cytoskeleton.

Exposed immune cells, showed levels of expression of the *Pl*-*NF-kB* gene 2-fold lower than those measured in controls (P_value_ = 0.02007) by comparative *q*PCR (Fig. 6A upper panel), while the transcripts of *Pl*-*Jun* showed a small increase (1.5-fold) (P_value_ = 0.00519) (Fig. 6A lower panel). Finally, we analysed by immunoblotting the intracellular protein levels of the interleukin-6 (IL-6), a cytokine known to be abundantly produced during the inflammatory phase, mainly (but not only) a direct target of the NF-*k*B transcription factor. The IL-6 was not detectable in any subcellular fraction of the sea urchin immune cells exposed to TiO_2_NPs. On the contrary, IL-6 was mostly detectable in the Cy and in the M/O fractions of control cells (Fig. 6B).

## Discussion

To our knowledge, this is the first study that investigates the effects of the *in vivo* exposure to TiO_2_NPs on immune cell of the sea urchin *Paracentrotus lividus.* To dissect the cellular and molecular mechanisms taking place, a combination of biological, molecular and biochemical approaches were applied. Overall results highlighted that TiO_2_NPs elicit a phagocytic mechanism carried out by phagocytes, involving the TLR/p38 MAPK signalling pathway. The geometry of particles has been recently recognised as an important parameter for biological functions such as phagocytic internalization, transport within the vascular system and blood circulation half-life^28^,^29^,^30^. In the human body, macrophages recognize size and shape of their targets, facilitating internalization *via* phagocytosis^31^,^32^. *Ex vivo* assays with organelle-specific dyes for live cell imaging showed that, the phagocytes of the sea urchin interact with TiO_2_NPs by activating an internalization mechanism. These cells have a dendritic-like phenotype and are the most abundant cell type present in the body cavity fluid of the sea urchin^5^. In a modest percentage of circulating phagocytes, we detected the progression of the phagosomal maturation (internalization, acidification, phagosomal-endosomal/lysosomal fusion) occurring from 10 to 90 minutes after *ex vivo* exposure to NR dye. Our findings are consistent with the notion that, as reported in macrophages exposed to small polystyrene particles (0.5 and 1 μm), a few cells can efficiently phagocytize a large number of smaller particles and simultaneously internalize a relatively large number of particles^32^. The modest percentage of TiO_2_NP-containing phagocytes suggest a change (renewal) in the phagocytic cell population as supported by the slight increase in the relative number of phagocytes, mantaining the homeostasis of the total cell number. Overall results provide the evidence that the TiO_2_NP uptake mechanism is generated by a receptor–ligand interaction even if, as established in mammalian macrophages, a non-specific passive uptake mechanism cannot be excluded depending on the size of particles^33^.

As well known, macrophages activate a wide range of PRR including TLRs, which enable the detection of conserved microbial cell wall component, such as LPS, lipoproteins and glycolipids^14^. For example, LPS induce TLR4 activation by a coordinate and sequential action of three other proteins (the lipopolysaccharide binding protein LBP, the cluster differentiation antigen CD14, the myeloid differentiation protein MD-2 receptors) that bind LPS in the monomeric form and present it, as a complex, to TLR4^34^. In this study, we found that TiO_2_NPs cause an increase in the expression of TLR gene and in the levels of the related protein in immune cells of *Paracentrotus lividus*. This result is in agreement with recent reports highlighting that TLR4 mediates the uptake of some engineered nanoparticles and small molecules in mammals, including TiO_2_NPs^34^,^35^,^36^.

This finding is not surprising, given that NP aggregates/agglomerates fall into the size, shape, geometry and surface chemistry of a wide range of bacteria and fungi^37^. Since immune cells first interact with the physical features of particles, these properties must necessarily have an important role in the recognition of the non-self-matter. Authors support the idea that small molecules and nanoparticles can bind directly the MD-2-TLR4 complex, excluding receptors (LBP, CD14) upstream the canonical LPS sequential recognition; alternatively, they can interfere in other points of the TLR4 signalling^34^. Typically, TLR4 activation triggers a signalling cascade leading to the activation of MAPKs (p38 MAPK, ERK, JNK), resulting in the increased expression and maintenance of a few pro-inflammatory cytokine genes, including IL-6, IL-12, TNF-α^38^. In this work, the unexpected interesting finding is that, contrary to what reported in the literature, TiO_2_NP-exposed cells displayed a significant inhibition in the p38 MAPK phosphorylated levels, together with the down-regulation of the *Pl*-*NF*-*k*B gene expression and the reduction of the IL-6 intracellular levels. ERK phosphorylation was not affected. Unfortunately, we cannot analyse the activation state of JNK, because commercially available antibodies recognizing the phosphorylated JNK did not cross-react with *Pl*-JNK. In general, phosphorylated JNK activates the AP-1 protein group composed of members of the Jun and Fos families, inducing the transcription of the complex independently of the *de novo* protein synthesis, as well as transcription of other target genes^39^. In this study, we demonstrated that *Pl*-*Jun* mRNA levels showed a weak increase in immune cells exposed to TiO_2_NPs, and, due to the known interaction between JNK and the AP-1 complex, we can hyposize that JNK activation could be barely involved in the mechanism(s) operating in response to TiO_2_NP exposure. For example, in macrophages it is known that the dexamethasone glucocorticoid selectively mediates the inhibition of p38 MAPK, but not inhibition of others MAPKs (e.g. PI3K/Akt, ERK, JNK)^40^. This result was directly correlated with the induction of the MAP kinase phosphatase-1 (MKP-1) achieved through the glucocorticoid receptors (GR). These receptors have been shown to be needed for macrophage survival after TLR4 expression, on the one hand to mitigate p38 MAPK signalling, and on the other hand to up-regulate JNK signalling by MKP-1 induction^40^. In agreement, a possible explanation for our results, including P-p38 MAPK/IL-6 inhibition/reduction, *Pl*-*NF-kB* down-regulation, and *Pl*-*Jun* up-regulation triggered by TiO_2_NP exposure, could be an increased activity of the MKP responsible for the complex negative regulatory mechanism that acts to control the duration, magnitude and spatial-temporal profile of the p38 MAPK and JNK activities. This mechanism could have an anti-inflammatory effect, contributing to avoid an over-reaction (inflammatory response) due to the engagement of TLR4-like, and to increase TiO_2_NP tolerance in the sea urchin immune cells^5^. In general, phagocytic activity is essential to maintain organs, such as intestine and lung, in a clean and sterile state. In the sea urchin exposed to TiO_2_NPs, the process of clearance seem to be complete within a day: in fact, the coelomic fluid inspected after immune cell collection did not show visible particles. Three explanations can be put forward: i) TiO_2_NPs are efficiently phagocytized and the host cells are cleared; ii) TiO_2_NPs move from the coelomic fluid to others part of the body and accumulate in other organs or tissues; iii) TiO_2_NPs are eliminated out of the body through the open circulatory system, the water vascular system^41^. Future studies in these directions are needed to clarify at least one of these intriguing hypotheses, although other plausible explanations cannot yet be excluded.

Reactive oxygen species (ROS) act through the p38 MAPK to limit the lifespan of mammalian hematopoietic stem cells, triggering cell-cycle arrest and senescence^42^,^43^. We found that in sea urchin immune cells, p38 MAPK is already activated under physiological conditions, likely to play a key role in determinig survival, terminal differentiation, proliferation, apoptosis and senescence of cells. Based on this evidence, we can hypothesize a possible involvement of the p38 MAPK pathway in the maintanance of stem cells homeostasis in the sea urchin (a few circulating stem cells in the body fluid), inhibiting self-renewing cell divisions and preventing the proliferation of potential cancer cells under physiological condition. In accordance, recent analysis of the protein carbonyl content in immune cells of three different sea urchin species with different lifespans, revealed that in the sea urchins modest physiological levels of intracellular ROS are required during the whole lifespan^44^, probably to maintain genomic stability through the activation of DNA repair mechanism. In the sea urchin no cases of cancer, immune and age-related diseases have been reported^45^. A fascinating hypothesis to link the results obtained through this work together with the findings that p38 MAPK is implicated in the control of the stem cell lifespan^42^, would suggest a role for TiO_2_NPs in the activation of the cell cycle progression and in the renewal of the sea urchin immune stem cells. Further studies in these directions are needed to confirm or reject this hypothesis.

For over a century, sea urchin has served as a model organism in developmental biology research, contributing to understand not only development and differentiation, but also to the origins of cellular and molecular biology^46^. Following the publication of the sea urchin genome^6^, a new prospect seems to be opened in biological research: the use of the sea urchin immune cells as a tool to uncover basic molecular and regulatory mechanisms of immune response, immune disease and immuno-toxicity in a *proxy to human* model. The value of this marine model for immune research is because many universal cellular properties are in common in all organisms. In addition, its plasticity to environmental changes^47^ should not be underestimated. For all those reasons, studies on sea urchin resistance to immune and age-related diseases and on tolerance to anthropogenic insults may contribute to highlight the key protective molecules, which could be used in innovative applications at the cutting edge of biomedicine. To the best of our knowledge, this is the first study describing immune response to nanoparticles in Echinoderms, as well as defining the potential molecular pathway(s) elicited *in vivo* by TiO_2_NPs in the sea urchin. The importance of our studies on sea urchin immune cells is two fold: i) to provide a new powerful model for nano-safety investigations as a proxy *to humans* and ii) to confirm themselves as an alternative model for ecotoxicological studies.

## Methods

### Animal handling and exposure to NPs

Adult sea urchins (*Paracentrotus lividus*) were collected along the Northwest coast of Sicily. They were maintained for several weeks under controlled conditions of temperature and salinity in oxygenated Artificial Seawater (ASW)(58.5% NaCl- 26.5% MgCl_2_-6H_2_O- 9.8% Na_2_SO_4_- 2.8% CaCl_2_- 1.65% KCl- 0.5% NaHCO_3_- 0.24% KBr- 0.07% H_3_BO_3_- 0.0095% SrCl_2_-6H_2_O- 0.007% NaF). Animals were fed every 7 days. TiO_2_NPs were sterilized under UV light, suspended in sterilized ASW, which was prepared according to standard protocols for eco-toxicological testing^21^, and sonicated for 1 hour at 70 W, 50% on/off cycle, in an ice/water bath. The suspension was diluted with ASW to 1 or 5 μg/ml TiO_2_NPs nominal concentration, passed through a sterile narrow opening syringe needle (30 gauge). Five hundred μl of the each diluted suspension was immediately injected into the sea urchin body cavity through the peristomial membrane surrounding the mouth. Immune cells were harvested as a total cell population (suspension), 24 hours after exposure, by bleeding of sea urchins through a cut in the peristomal membrane, in an anticoagulant solution, namely coelomocyte culture medium (CCM), (2× CCM), as previously described^48^. Specimens were obtained from three independent experiments: three sea urchins for each of the two different TiO_2_NP concentrations and three controls (injected only with ASW) for each experiment.

In order to avoid false readouts linked to the experimental approach, positive controls were included in the experiments. To this purpose, a few sea urchins were injected with lipopolysaccharide (2 μg/ml in ASW) (LPS, Sigma Aldrich, St. Louis, MO). All injected sea urchins were maintained in dedicated aquaria (20 litres) with running SW and oxygenation at 18 °C for 24 hours (3 sea urchins/tank). The commercial Aeroxide P25 Titanium Dioxide powder (declared particle size: 21 nm) was obtained from Evonik Degussa (Essen, Germany). TiO_2_NPs dispersed in ASW were previously characterized by a combination of analytical techniques (transmission electron microscopy, Brunauer, Emmett and Teller method, dynamic light scattering), as described in Brunelli *et al.* 2013.

### Immune cells handling and immunoblotting analyses

Approximately, five ml of coelomic fluid (CF), containing freely circulating immune cells, were poured on 5 ml ice-cold 2× CCM, composed of 1 M NaCl, 10 mM MgCl_2_, 40 mM Hepes, 2 mM EGTA pH 7.2^48^. After collection in the anticoagulant solution, immune cells were counted in a Fast-Read chamber (Biosigma) and the Trypan blue exclusion test was used to determine the number of viable cells present in the cell suspension as total cell population. The cell suspension was immediately centrifuged at 9000 g for 5 minutes and pellets homogenized in a lysis buffer^49^ supplemented with a protease inhibitor cocktail (Roche) and phoshatase inhibitor cocktail (SIGMA). Cytosolic, membrane/organelle, nuclear, and cytoskeletal fractions were prepared using a ProteoExtract subcellular proteome extraction kit (Calbiochem, Merck), according to the manufacturer’s instructions, with minor changes. The protein contents of the extracts were quantified by the Bradford method with the BioRad (Hercules, CA, USA) assay kit. Fifteen micrograms of total protein per each cell extract and twenty micrograms of protein per each subcellular fraction were run on a 4–15% Mini-PROTEAN TGX precast polyacrylamide gels (Bio-Rad) and transferred to nitrocellulose membranes (Amersham), according to standard procedures. Non-specific binding sites were blocked with Odyssey blocking buffer (LI-COR Biosciences), for 1 h at room temperature (RT). After blocking, replicate membranes were incubated overnight at 4 °C with either one of the following primary antibodies in Odyssey blocking buffer-0.1% Tween20: i) Phospho-p38 MAP Kinase (Thr180/Tyr182) (Cell Signaling, 9211) 1:250; ii) p38 MAP Kinase non-activated form (SIGMA, M8432) 1:500; iii) Phospho-p42/44 MAP Kinase (ERK1/2) (Cell Signaling, 9101) 1:300; iv) TLR4 (H-80) protein (Santa Cruz Biotechnology, sc-10741) 1:250; v) IL-6(H-183) (Santa Cruz Biotechnology, sc-7920) 1:100; vi) HSP70 (SIGMA, Cat N. H-5147) 1:3000. Membranes were washed three times with PBS-Tween20 prior to incubation with a fluorescein-labelled secondary anti-mouse and/or -rabbit antibody (LI-COR Biosciences). Protein levels were normalized with actin or tubulin levels (assumed constant) determined by the use of an Anti-β-actin (SIGMA, A5441) or Anti-α-tubulin (SIGMA, T5168) on the same filters. Results were reported in arbitrary units obtained from the volumetric analysis of the normalized bands (sum of the three independent experiments).

### *Ex vivo* assays

Live immune cells were incubated at RT in 40 μg/ml NR dye for 15 minutes (200 μl cell suspension/tube). As NR is a pH-sensitive dye used to follow the pH change of acid vesicles in living cells^50^, we used it as marker of phagosome acidification (phagolysosome formation) in phagocytic cells. Staining of the internal membranes was performed by the use of 2.5 μg/ml DiOC_6_, a green fluorescent membrane dye which at high concentrations, binds to the endoplasmic reticulum, Golgi and vesicle membranes^51^. Immune cells were observed with a Zeiss Axioskop 2 Plus microscope (Zeiss, Arese, Italy), equipped for epifluorescence, and recorded by a digital camera.

### RNA extraction, cDNA synthesis, cloning and sequencing

Total RNA from control and exposed immune cells was isolated, according to Russo *et al.*^52^, from specimens obtained from three independent experiments. Briefly, total RNAs from each sample (1 μg) were reverse transcribed according to the Applied Biosystems manufacturer’s instructions (Applied Biosystems, Life technologies, Carlsbad, CA, USA). Twenty ng of each cDNA was amplified by Polymerase Chain Reaction (PCR), the amplicons cloned in the pGEM-Teasy vector (PROMEGA, Madison, WI, USA), and sequenced by a service company (BIO-FAB research srl, Rome). The isolated and sequenced nucleotide fragments were searched using the Basic Local Alignment Search Tool (BLAST, NCBI) and sequences have been deposited at NCBI^53^ under the following accession numbers: LK022847 (*Pl*-*TLR*); LK022846 (*Pl*-*p38 MAPK*); HE574572 (*Pl*-*NF-kB*) and HE817756 (*Pl*-*Jun*) (Table 2). The last two sequences have been previously identified in embryos^54^,^55^ and, after sequence analysis of amplicons obtained from immune cells, confirmed to be identical.

### Real-time *quantitative PCR (qPCR)*

To quantify the expression of immune responsive genes (*TLR, p38 MAPK, NF-kB, Jun*), the cDNAs isolated from control and TiO_2_NPs exposed *Paracentrotus lividus* immune were amplified by SYBR Green based real-time *q*PCR, as described in the manufacturer manual (Applied Biosystems Step One Plus real time PCR, a Comparative Threshold Cycle Method)^56^. The *Pl-Z12-1* mRNA, encoding a zinc-finger transcription factor, was used as the internal endogenous reference gene^57^. The primer sequences used for real-time *q*PCRs and the amplicon lenghts (ranging from 50 to 150 nt) for the selected genes are summarized in Table 2. The real-time *q*PCRs were run as follows: 1× cycle denaturing 95 °C for 10 minutes and 38× cycles melting 95 °C for 15 second *plus* annealing/extension 60 °C for 60 second.

### Statistical analysis

All data were analyzed by the one-way analysis of variance (one-way ANOVA) compared with the respective control group, followed by the multiple comparison test of Tukey’s, using the OriginPro 7.5 statistical program with the level of significance set to P < 0.05. Each result is reported as the mean of three independent replicate experiments ±SE.

## Additional Information

**How to cite this article**: Pinsino, A. *et al.* Titanium dioxide nanoparticles stimulate sea urchin immune cell phagocytic activity involving TLR/p38 MAPK-mediated signalling pathway. *Sci. Rep.* **5**, 14492; doi: 10.1038/srep14492 (2015).

## Figures and Tables

**Table 1:** Characterization of Aeroxide© n-TiO_2_ P25 in ASW standard suspension.

Parameters	Techniques	Data
Primary size (nm)	TEM	10 to 65 nm
Shape		Irregular and Semi-spherical
Crystallographic phases		Anatase and Rutile (4:1)
Surface area	BET	61 m^2^/g
Pore size		0.5 ml/g
Structure		Mesoporous
Agglomeration (nm) in	DLS	350 ± 41 nm at 0.20 h
ASW suspension		466 ± 9 nm at 25 h
(1 μg/ml)		519 ± 59 nm at 50 h

Modified from Brunelli *et al.* 2013.

TEM: transmission electron microscopy.

BET: method of Brunauer, Emmett and Teller^58^.

DLS dynamic light scattering.

Anatase and Rutile: two mineral forms of the titanium dioxide.

**Table 2:** Primers used for *q*PCR and cDNA amplicon lengths.

Gene	Fw 5′- 3′	Rev 5′- 3′	Amplicon size nt	Accession number
*Pl-TLR*	ACTGTGATTTGGAGTGGTTTAT	AGGATCAAACTCAAGAAGGGGTT	128	LK022847
*Pl-NF-kB*	TCCCATGGAGGACTGCCGTGTCA	TCGTTGGTTACCAAGGAGACCACA	116	HE574572
*Pl-p38 MAPK*	TTCACTGCCAGAGGACTTCCATCA	ATACTTGCCCATACGCTCCCGA	110	LK022846
*Pl-Jun*	GAGACTCAGTTCTACGAAGATTCA	AGGCAAGCTTGAGCATCTGTACGT	139	HE817756

*Pl-TLR* region: Leucine rich repeat C-terminal domain.

*Pl-NF-kB* region: N-terminal sub-domain of the Rel homology domain (RHD).

*Pl-Pl-p38 MAPK* region: Catalytic domain of the Protein Kinase superfamily.

*Pl-Jun* region: Jun-like domain.
